# The influence of family cultural capital on student learning engagement: a study on the mediating role of parental educational involvement

**DOI:** 10.3389/fpsyg.2026.1802893

**Published:** 2026-04-10

**Authors:** Juanjuan Zang, Pengfei Chen

**Affiliations:** Department of Pre-School Education, School of Education, Shandong Women’s University, Jinan, Shandong, China

**Keywords:** family cultural capital, latent profile analysis, learning engagement, mediating effect, parental educational involvement

## Abstract

**Introduction:**

In the digital era, the role of family in education is increasingly prominent. While cultural capital theory offers a framework for understanding educational inequality, the dynamic conversion of new forms of family cultural capital, such as digital resources, into student learning engagement remains underexplored. This study addresses this gap by examining the mediating role of parental educational involvement in this process, providing a nuanced understanding of how family resources translate into academic advantages.

**Methods:**

This study utilized a questionnaire survey method, collecting data from 610 parents of primary and middle school students in Eastern China. The scales for Family Cultural Capital (FCC), Parental Educational Involvement (PEI), and Student Learning Engagement (LE) demonstrated good reliability and validity. The data were analyzed using structural equation modeling, the Bootstrap method for mediation testing, latent profile analysis (LPA), and multi-group analysis.

**Findings:**

The results indicated that: (1) Family cultural capital has a significant positive predictive effect on student learning engagement (*β* = 0.42, *p* < 0.001). (2) Parental educational involvement partially mediates this relationship, with the mediating effect accounting for 45.1% of the total effect. (3) Latent profile analysis identified three distinct family profiles: “Resource-Poor,” “Digital Emerging,” and “All-Round.” (4) The relationship between variables is moderated by urban–rural differences and grade level. Specifically, the conversion of cultural capital into parental involvement is more efficient in urban families, while the impact of parental involvement on learning engagement is stronger in rural families. For lower-grade students, the effect of cultural capital is primarily mediated by parental involvement, whereas for higher-grade students, the direct influence of cultural capital becomes more prominent.

**Discussion:**

The findings extend Bourdieu’s cultural capital theory by incorporating digital capital and revealing the dynamic, context-dependent nature of its influence. The study underscores the critical role of active parental involvement as a mechanism for resource conversion and highlights how this process varies across different social contexts and developmental stages. These insights offer valuable guidance for parents, schools, and policymakers aiming to foster educational equity.

## 1 Introduction

### 1.1 Research background

Learning engagement stands as a barometer for academic achievement and long-term vitality. Beyond immediate grades, it shapes mental resilience and lifelong adaptability (Wong et al., 2024). Against the backdrop of China’s “Double Reduction” policy, which compresses school hours, the locus of educational responsibility has shifted perceptibly toward the family. A shift that has been linked to heightened parental educational anxiety and a stronger perception of educational involution (Zhang et al., 2024).

Bourdieu’s (1986) cultural capital theory provides a theoretical framework for understanding the intergenerational transmission of family educational advantages. Cultural capital includes the objectified state (e.g., books, artworks), the embodied state (e.g., knowledge, skills), and the institutionalized state (e.g., academic credentials). Traditional research has mostly focused on the impact of physical cultural resources (such as book collection size) on academic achievement, but in the digital age, the forms of cultural capital are undergoing profound changes. Digital devices, online learning resources, and information literacy have become new forms of cultural capital (Ragnedda, 2018).

However, existing research has three shortcomings: first, most studies view cultural capital as a static resource, ignoring the dynamic process of its conversion into student learning behaviors (e.g., Sullivan, 2001, often focuses on the correlation between capital stock and outcomes rather than the process); second, empirical testing of the key mediating mechanism of parental educational involvement is insufficient, leaving the “how” of capital conversion under-theorized (Fan and Williams, 2010); third, there is a lack of systematic measurement of new forms of cultural capital in the digital age, as many scales still prioritize traditional indicators like book counts (Yang et al., 2022).

### 1.2 Research purpose and significance

This study aims to construct a theoretical model of “Family Cultural Capital → Parental Educational Involvement → Student Learning Engagement” and test the validity of this model through empirical data. The theoretical significance of the study lies in: (1) expanding the applicability of cultural capital theory in the digital age; (2) revealing the mediating mechanism by which cultural capital affects learning engagement; (3) providing empirical evidence for educational equity policies. The practical significance lies in providing a scientific basis for family education guidance, home-school cooperation, and educational resource allocation.

## 2 Literature review and research hypotheses

### 2.1 Theoretical framework: cultural capital theory

This study is grounded in Pierre Bourdieu’s (1986) cultural capital theory. Bourdieu defines cultural capital as the collection of symbolic elements such as skills, tastes, posture, clothing, mannerisms, material belongings, credentials, etc. that one acquires through being part of a particular social class. He distinguishes between three states of cultural capital:

1. The embodied state: capital incorporated within the individual, such as knowledge, dispositions, and skills. It is acquired over time and cannot be transmitted instantaneously.
2. The objectified state: capital held in the form of cultural goods, such as books, paintings, or scientific instruments. These can be transferred for economic profit.
3. The institutionalized state: capital recognized in the form of academic qualifications or credentials, which provides a legally guaranteed value.

This theory informs the current study by providing a lens to understand how non-economic family resources (cultural capital) contribute to educational inequality. The study posits that the “conversion” of objectified cultural capital (e.g., books, digital resources) into children’s embodied capital (e.g., learning engagement) is not automatic. Instead, it is a dynamic process actively facilitated by parents. This study operationalizes this facilitation process as “parental educational involvement,” treating it as the key mechanism through which the potential of family cultural capital is actualized.

### 2.2 Operational definitions of core concepts

To ensure clarity, the core concepts of this study are operationally defined as follows:

Family Cultural Capital (FCC): Drawing from Bourdieu’s framework and its modern interpretations (Ren et al., 2022), FCC in this study refers to the sum of cultural resources available within a family that can support a child’s education. It is measured across three dimensions: (1) Physical Cultural Resources (e.g., number of books, dedicated learning space), (2) Digital Cultural Resources (e.g., subscription to online learning platforms, parental guidance on using digital tools), and (3) Community Cultural Participation (e.g., visits to museums and libraries).

Parental Educational Involvement (PEI): Based on Epstein’s (2010) model, PEI is defined as the specific actions parents take to support their child’s learning. It encompasses two dimensions: (1) Home Learning Support (e.g., discussing school life, accompanying reading) and (2) Home-School-Community Connection (e.g., communicating with teachers, participating in social practice activities).

Student Learning Engagement (LE): Following the multidimensional framework by Fredricks et al. (2004), LE is defined as the extent to which a student is actively involved in their learning process. As observed by parents, it includes three dimensions: (1) Behavioral Engagement (e.g., task completion, persistence), (2) Emotional Engagement (e.g., curiosity, enjoyment), and (3) Cognitive Engagement (e.g., making connections between concepts, self-reflection).

### 2.3 Family cultural capital and student learning engagement

Cultural capital theory posits that cultural resources possessed by families influence children’s educational achievement through a “cultural transmission” mechanism (Bourdieu, 1986). Empirical studies show that family book collections and the frequency of participation in cultural activities are positively correlated with students’ academic performance and cognitive abilities (Sullivan, 2001; Yang et al., 2022).

In recent years, researchers have begun to focus on the impact of cultural capital on learning engagement. Learning engagement is a multidimensional construct, including behavioral engagement (e.g., attendance, homework completion), emotional engagement (e.g., learning interest, sense of belonging), and cognitive engagement (e.g., deep learning strategies, metacognition) (Fredricks et al., 2004). A longitudinal study by Chen et al. (2024) found that family cultural capital can predict students’ emotional and cognitive engagement as early as the primary school stage.

In the digital context, the connotation of cultural capital is expanding. Pitzalis and Porcu (2024) deeply deconstructed the intersection of digital capital and cultural capital, arguing that digital devices and digital literacy have become core constructs in family resource allocation. Research shows that digital cultural capital has a unique predictive effect on students’ online learning engagement, and this effect is independent of traditional cultural capital (Ren et al., 2022).

Based on the above literature, Hypothesis 1 is proposed:

*H1*: Family cultural capital has a significant positive predictive effect on student learning engagement.

### 2.4 The mediating role of parental educational involvement

Parental educational involvement refers to the time, energy, and resources parents invest in their children’s education (Epstein, 2010). Existing research indicates that parental educational involvement is a key bridge connecting family resources and student development (Fan and Williams, 2010). Empirical studies on Chinese adolescents also found that parental educational involvement can significantly improve students’ academic engagement levels (Zhu et al., 2023).

Theoretically, cultural capital is associated with parental involvement through two paths: first, the resource path—families with more cultural resources are more capable of providing high-quality learning support; second, the value path—families rich in cultural capital tend to value education more, and parents are more willing to invest time in participating in their children’s education (Lareau, 2018). In the highly competitive Chinese context, this model of “concerted cultivation” often manifests as intense parental strategies and substantial investment in “shadow education” to secure a competitive edge for their children (Zhang, 2020).

Empirical research supports this mediating relationship. In a longitudinal study of 1,000 primary school students, Zhang et al. (2020) found that parental educational involvement fully mediated the impact of family socioeconomic status on student academic achievement. Research in the Chinese context shows that parental home tutoring and home-school communication behaviors are key mechanisms for converting cultural capital into student learning motivation.

Based on the above analysis, Hypothesis 2 and Hypothesis 3 are proposed:

*H2*: Parental educational involvement has a significant positive association with student learning engagement.

*H3*: Parental educational involvement plays a mediating role in the relationship between family cultural capital and student learning engagement.

### 2.5 Analysis of moderating effects

There are significant differences in educational resources between urban and rural areas in China. Urban families are superior to rural families in economic capital, cultural capital, and social capital (Xie and He, 2023; Tan and Fang, 2023). Such differences may moderate the effect of cultural capital.

The compensation hypothesis suggests that in resource-poor environments, limited cultural capital may play a larger role (Roscigno and Ainsworth-Darnell, 1999). Conversely, the cumulative advantage hypothesis suggests that resource-rich environments can amplify the effects of cultural capital (DiPrete and Eirich, 2006).

Research has found that in rural areas of China, the impact of family cultural capital on student academic achievement is weaker, which may be because the lower quality of rural schools weakens the role of family resources (Ren et al., 2022). However, studies also show that in rural areas, the impact of parental educational involvement on student development is stronger because the lack of school support makes the family’s role more prominent.

Given these conflicting findings, competitive hypotheses are proposed:

*H4*: Urban–rural differences moderate the influence of family cultural capital on parental educational involvement.

In addition to the social environment, the developmental stage of students is also an important factor affecting the effectiveness of family education. As students grow older, their autonomy increases and their cognitive abilities improve. Their reliance on direct parental behavioral involvement (such as homework tutoring) may decrease, while they may become more influenced by the direct edification of the family cultural environment (such as book collections, atmosphere). This means that the path of cultural capital’s effect may change dynamically with grade level.

Based on this, the following hypothesis is proposed:

*H5*: Student grade level moderates the influence path of family cultural capital on student learning engagement.

**Figure 1:**
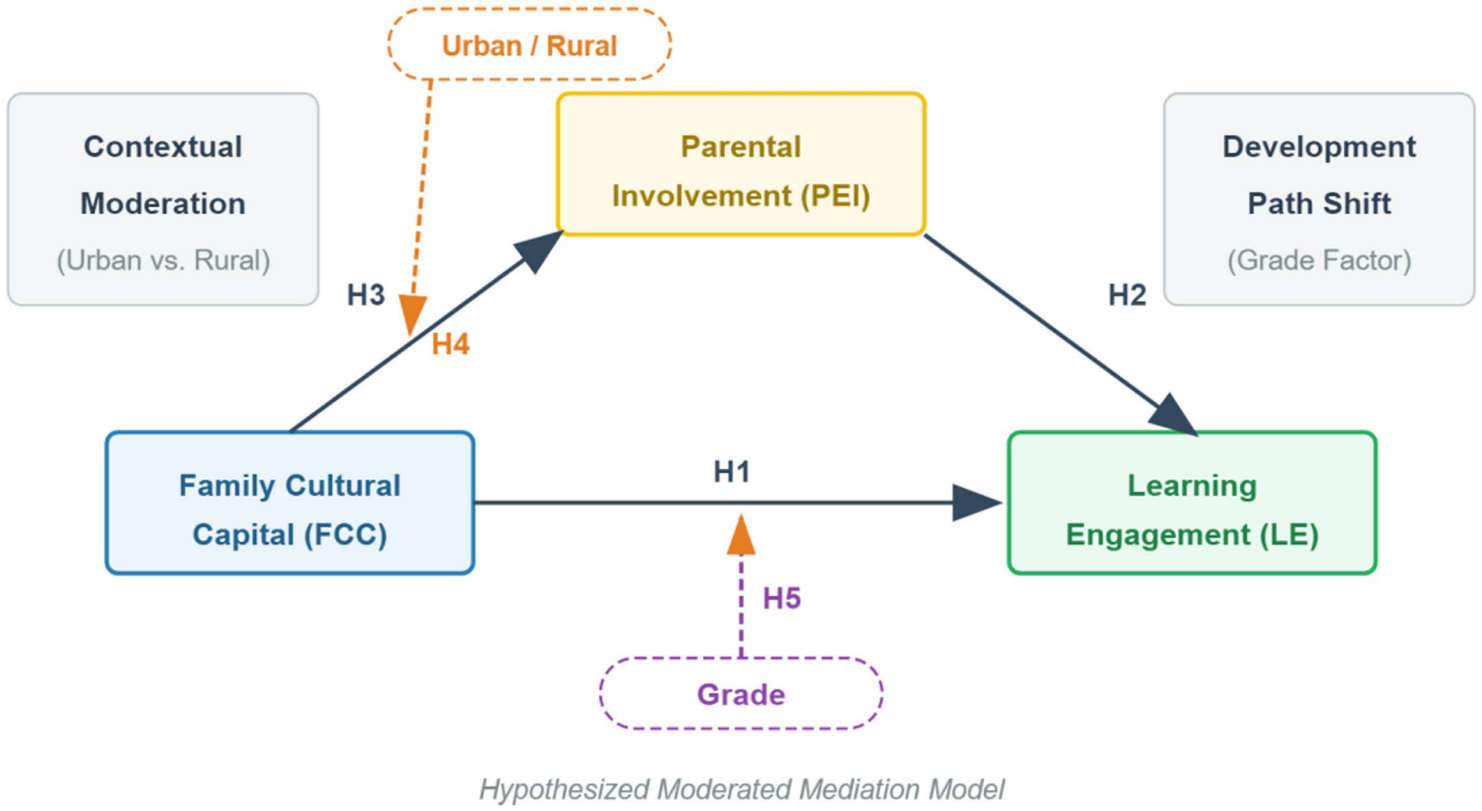
Theoretical framework model of the research.

Based on the discussion of the relationships between family cultural capital, parental educational involvement, urban–rural differences, and grade level, this study integrates and constructs a moderated mediation model. This model not only examines the mediating path of cultural capital conversion into learning engagement but also thoroughly explores the moderating effects of the environment (urban/rural) and individual developmental stage (grade) on different links, as shown in Figure 1.

## 3 Research methods

### 3.1 Participants and procedure

This study adopted a convenience sampling method and conducted a questionnaire survey in Eastern China (Jiangsu, Zhejiang, Shanghai) from September to November 2025. Questionnaires were distributed through school parent groups and online platforms. An informed consent form was set on the first page of the questionnaire, clarifying that the data would be used only for academic research and kept strictly confidential. This study intentionally included participants from both primary (grades 1–6) and secondary (grades 7–9) schools. This cross-sectional approach across different grade levels was chosen to facilitate an exploration of how the relationships between family cultural capital, parental involvement, and student engagement might differ across key developmental stages, which is a central component of our moderated mediation model (see Hypothesis 5).

Data cleaning criteria were as follows: (1) Samples with a response time of less than 60 s were excluded; (2) Samples showing regular response patterns (such as selecting the same option for all) were excluded; (3) Samples that failed to select the correct option in the attention check question were excluded. Finally, 610 valid questionnaires were obtained, with an effective recovery rate of 93.8%.

The sample was mainly filled out by mothers (68.9%), with a balanced distribution of family locations (Urban 31.1%, Town 35.2%, Rural 33.8%), and student grades covering primary to junior high school (Lower Primary 28.5%, Upper Primary 35.7%, Junior High 35.7%).

### 3.2 Research tools

#### 3.2.1 Family cultural capital scale (FCC)

Adopting Bourdieu’s (1986) classification framework, including physical cultural resources (e.g., books) and social participation dimensions, and incorporating digital cultural resource items (2 items) based on the research of Ren et al. (2022), the scale contains 7 items covering three dimensions: physical cultural resources (book volume, learning space), digital cultural resources (digital resource subscription, digital device guidance), and community cultural participation (museum visits, community activities, use of cultural facilities). Among them, the item “independent learning space” under the “Physical Cultural Resources” dimension uses binary scoring (0 = No, 1 = Yes), and the remaining items use a Likert 5-point scale. In this study, Cronbach’s *α* = 0.82. Confirmatory factor analysis showed that the three-factor model (i.e., physical resources, digital resources, community participation) fit well (*χ*^2^/*df* = 2.31, CFI = 0.96, TLI = 0.94, RMSEA = 0.046), indicating that the mixed scoring format did not affect the valid measurement of the latent construct. Specific items are shown in Appendix A.

#### 3.2.2 Parental educational involvement scale (PEI)

Compiled with reference to Epstein’s (2010) model, containing 6 valid items (another item was an attention check question, excluded during statistical analysis), divided into two dimensions: home learning support (discussing school situations with children, creating a learning environment, reading accompaniment) and home-school-community connection (communicating with teachers, attending parent-teacher meetings, social practice). A Likert 5-point scale was used. Cronbach’s *α* = 0.84, and the two-factor model fit well (*χ*^2^/*df* = 2.18, CFI = 0.97, TLI = 0.95, RMSEA = 0.044). Specific items are shown in Appendix B.

#### 3.2.3 Student learning engagement scale (LE)

Adopting the three-dimensional framework proposed by Fredricks et al. (2004) and referring to the empirical scale of Wang and Eccles (2012), a parent observation version was compiled containing 7 items, covering three dimensions: behavioral engagement (completing tasks with self-discipline, persistence in studying), emotional engagement (curiosity, enjoyment), and cognitive engagement (linking knowledge, self-reflection, application of knowledge). A Likert 5-point scale was used. Cronbach’s *α* = 0.86, and the three-factor model fit well (*χ*^2^/*df* = 2.42, CFI = 0.96, TLI = 0.94, RMSEA = 0.048). Specific items are shown in Appendix C.

#### 3.2.4 Control variables

Student gender (1 = Male, 2 = Female), grade (1 = Lower Primary, 2 = Upper Primary, 3 = Junior High), only child status (1 = Yes, 2 = No), and family socioeconomic status (SES, synthesized by taking the mean of Z-standardized scores of parents’ highest education and family monthly income) were controlled.

### 3.3 Data analysis strategy

SPSS 26.0 was used for descriptive statistics and correlation analysis. Harman’s single-factor test was used to test for common method bias. AMOS 24.0 was used for confirmatory factor analysis (CFA) and structural equation modeling analysis. The PROCESS Macro (Model 4, Bootstrap = 5,000) was used to test the mediation effect. Mplus 8.3 was used for latent profile analysis (LPA). Multi-group structural equation modeling was used to test the moderating effect. The significance level was set at *α* = 0.05. The common method bias test result showed that the variance explained by the first principal factor was 28.43% (<40%), and the three-factor model was significantly better than the single-factor model (Δ*χ*^2^ = 1245.67, *p* < 0.001), indicating that this study was not affected by serious common method bias (see Table 1).

**Table 1:** Basic characteristics of the sample (*N* = 610).

Variable	Category	Count	Percentage (%)
Respondent	Mother	420	68.9
	Father	190	31.1
Region	Urban	190	31.1
	Town	215	35.2
	Rural	206	33.8
Grade	Lower primary	174	28.5
	Upper primary	218	35.7
	Junior high	218	35.7

## 4 Research results

### 4.1 Descriptive statistics and correlation analysis

The means, standard deviations, and correlation coefficients of the variables are shown in Table 2. All core variables were significantly positively correlated (*r* = 0.42–0.55, *p* < 0.001), providing a statistical basis for subsequent mediation effect testing. SES was significantly positively correlated with all three core variables, supporting the necessity of using it as a control variable. Student grade was negatively correlated with parental educational involvement and learning engagement, indicating that as the grade increases, parental involvement and student engagement tend to decline.

**Table 2:** Descriptive statistics and correlation matrix of main variables (*N* = 610).

Variable	M	SD	1	2	3	4	5	6
1. Student gender	1.48	0.50	—					
2. Student grade	2.07	0.80	0.03	—				
3. SES	0.00	1.00	−0.02	0.05	—			
4. Family cultural capital	3.42	0.82	−0.04	−0.12**	0.51***	(0.82)		
5. Parental Edu. involvement	3.65	0.79	0.08*	−0.18***	0.43***	0.48***	(0.84)	
6. Student learning engagement	3.58	0.85	0.11**	−0.21***	0.39***	0.42***	0.55***	(0.86)

Cronbach’s *α* coefficients are in parentheses on the diagonal. ***p < 0.001, **p < 0.01, *p < 0.05 (two-tailed test).

### 4.2 Mediation effect test

**Figure 2:**
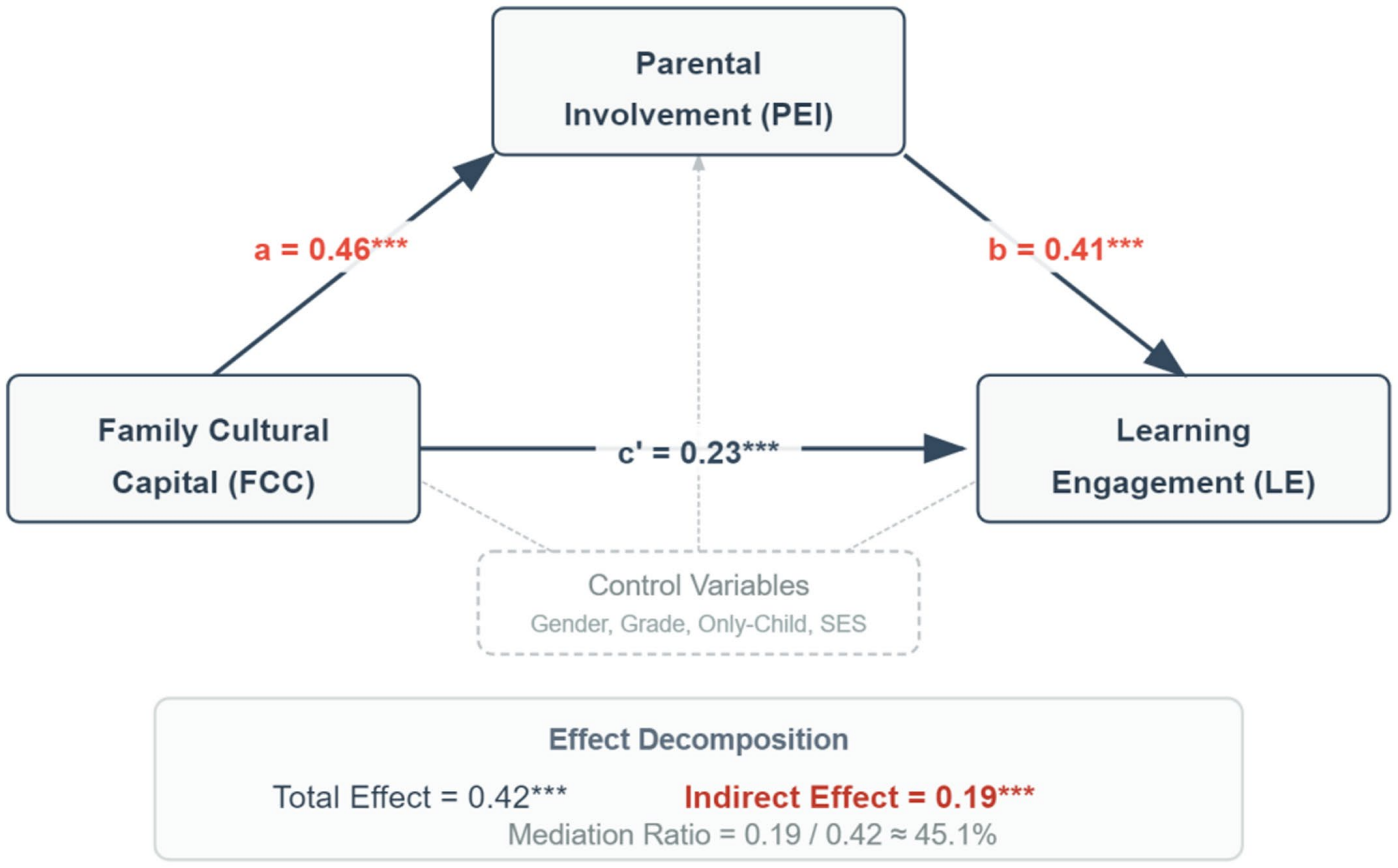
Path diagram of the mediating effect of parental educational involvement.

After controlling for student gender, grade, only child status, and family SES, PROCESS Model 4 was used to test the mediating role of parental educational involvement. The analysis results are shown in Table 3 and Figure 2.

**Table 3:** Test of mediating effect of parental educational involvement (*N* = 610).

Predictor	Outcome: parental edu. involvement	Outcome: student learning engagement
	*β* (SE)	*β* (SE)
Control variables		
Student gender	−0.03 (0.06)	0.07* (0.06)
Student grade	−0.11** (0.04)	−0.13** (0.04)
Only child status	0.05 (0.06)	0.02 (0.05)
SES	0.20*** (0.03)	0.15*** (0.03)
Independent variable		
Family cultural capital	0.46*** (0.04)	0.23*** (0.04)
Mediator		
Parental edu. involvement	—	0.41*** (0.04)
*R* ^2^	0.34	0.45
*F*	62.45***	74.23***

Standardized regression coefficients are reported. ***p < 0.001, **p < 0.01, *p < 0.05.

The results show: (1) Family cultural capital significantly positively predicts parental educational involvement (*β* = 0.46, *p* < 0.001); (2) After controlling for family cultural capital, parental educational involvement significantly positively predicts student learning engagement (*β* = 0.41, *p* < 0.001); (3) After adding the mediating variable, the direct effect of family cultural capital on learning engagement remains significant (*β* = 0.23, *p* < 0.001), but the coefficient dropped from the total effect of 0.42 to 0.23.

The bias-corrected Bootstrap method was used to further test the significance of the mediation effect, as shown in Table 4.

**Table 4:** Bootstrap test results of mediation effect.

Effect type	Effect value	Boot SE	95% CI lower	95% CI upper	Relative effect size
Total effect (c)	0.419	0.038	0.345	0.493	100%
Direct effect (c′)	0.230	0.041	0.150	0.310	54.9%
Indirect effect (a × b)	0.189	0.024	0.145	0.238	45.1%

Bootstrap sample size = 5,000. If the 95% confidence interval does not contain 0, the effect is significant.

The test results indicate that the indirect effect is significant (95% CI [0.145, 0.238]), accounting for 45.1% of the total effect. This supports H3, that parental educational involvement plays a partial mediating role between family cultural capital and student learning engagement.

### 4.3 Latent profile analysis

To deeply understand the heterogeneity of family cultural capital, Latent Profile Analysis (LPA) was used to classify 610 families. Using the 7 items of the FCC scale as indicator variables, models with 2–5 profiles were fitted sequentially. The model fit indices are shown in Table 5.

**Table 5:** Model fit indices for latent profile analysis.

No. of profiles	AIC	BIC	aBIC	Entropy	LMRT (*p*)	BLRT (*p*)
2	8245.67	8312.45	8256.89	0.82	<0.001	<0.001
3	8012.34	8098.23	8025.67	0.85	<0.001	<0.001
4	7998.45	8103.56	8014.23	0.79	0.082	<0.001
5	8005.67	8129.89	8023.45	0.76	0.156	<0.001

AIC, Akaike information criterion; BIC, Bayesian information criterion; aBIC, adjusted BIC; Entropy, entropy index; LMRT, Lo–Mendell–Rubin test; BLRT, bootstrap likelihood ratio test.

**Figure 3:**
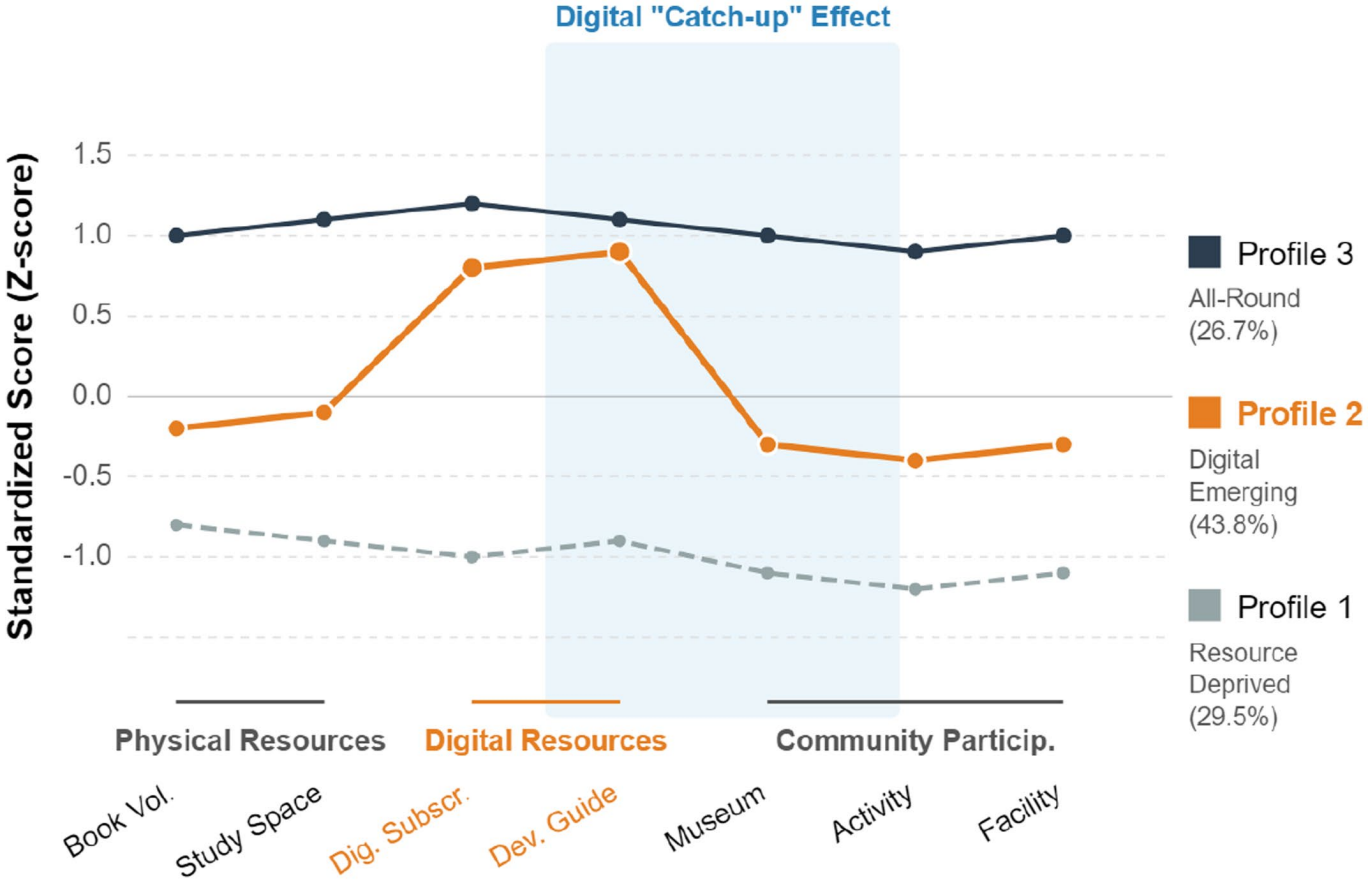
Three latent profiles of family cultural capital.

Considering model fit indices, theoretical interpretability, and sample distribution, the 3-profile model was selected. The characteristics of the three profiles are shown in Figure 3 and Table 6.

**Table 6:** Characteristic description of three latent profiles.

Profile	*N*	%	Main characteristics	FCC mean	PEI mean	LE mean
Profile 1: resource-poor	180	29.5%	Low scores on all dimensions, mainly rural low-income families	2.05	2.98	2.87
Profile 2: digital emerging	267	43.8%	Rich digital resources, moderate physical resources, mainly town middle-income families	3.45	3.78	3.72
Profile 3: all-round	163	26.7%	High scores on all dimensions, mainly urban high-income families	4.73	4.35	4.21

ANOVA results showed significant differences among the three profiles in parental educational involvement and student learning engagement (*F* = 145.67, *p* < 0.001; *F* = 132.45, *p* < 0.001). Post hoc comparisons (Bonferroni correction) indicated profile 3 > profile 2 > profile 1 (all *p* < 0.001).

**Figure 4:**
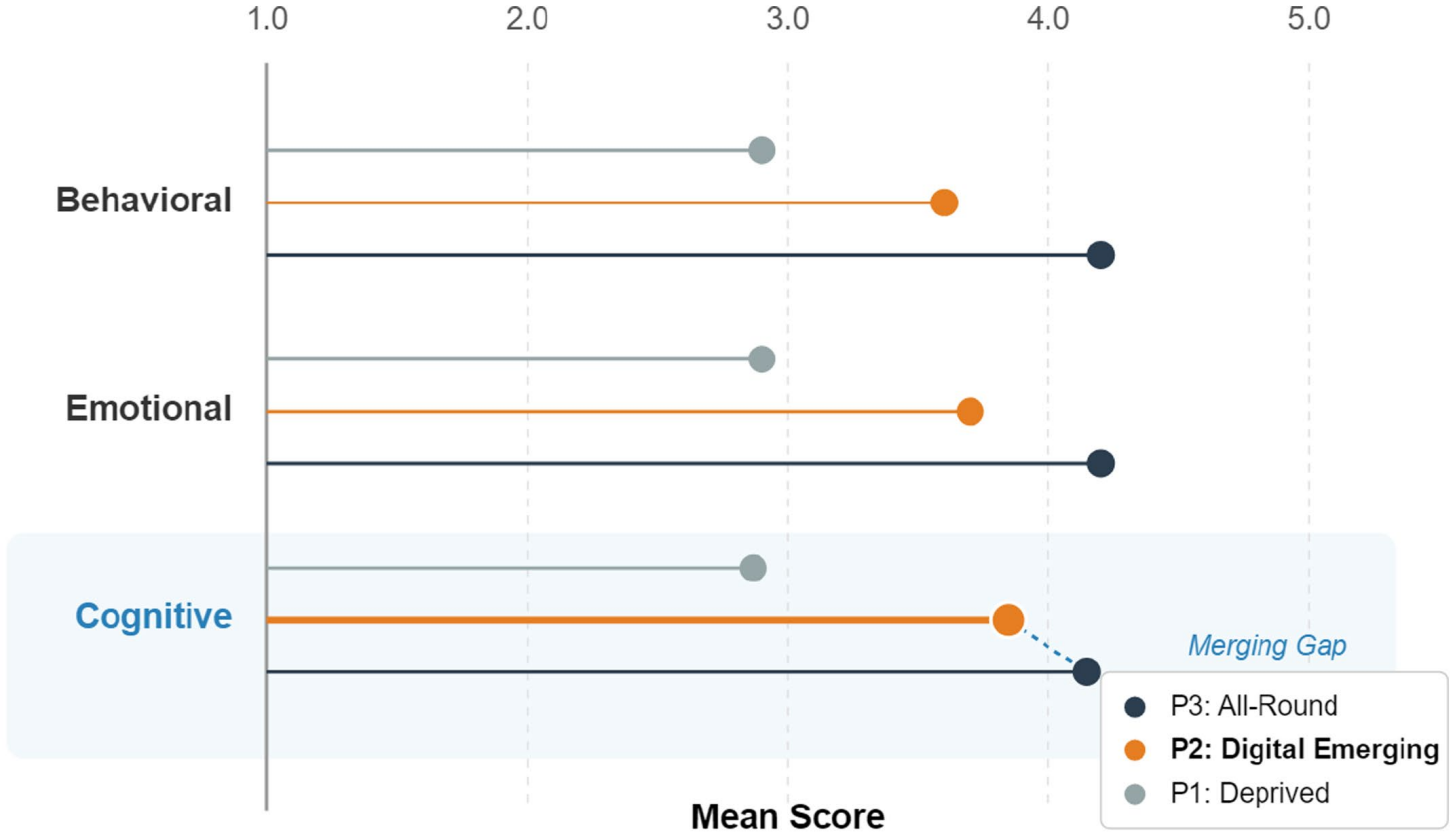
Comparison of scores on learning engagement dimensions across three profiles.

Further analysis of the three sub-dimensions of learning engagement, as shown in Figure 4, revealed that Profile 2 (Digital Emerging) scored significantly higher on the cognitive engagement dimension (M = 3.85) than on behavioral engagement (M = 3.62) and emotional engagement (M = 3.69), and was close to Profile 3’s score on cognitive engagement (M = 4.15). This indicates that digital cultural capital plays a unique role in cultivating students’ higher-order thinking skills.

### 4.4 Moderating effect analysis

#### 4.4.1 Moderating effect of urban–rural differences

To test whether urban–rural differences moderate the influence of family cultural capital on parental educational involvement, the sample was divided into an urban group (*n* = 190) and a rural group (n = 206) for multi-group structural equation modeling analysis. First, measurement invariance was tested, and results showed that both configural invariance (*χ*^2^/*df* = 2.18, CFI = 0.95, RMSEA = 0.044) and metric invariance (Δ*χ*^2^ = 12.45, *p* = 0.132) held, indicating that the scale has the same measurement meaning in both groups.

**Figure 5:**
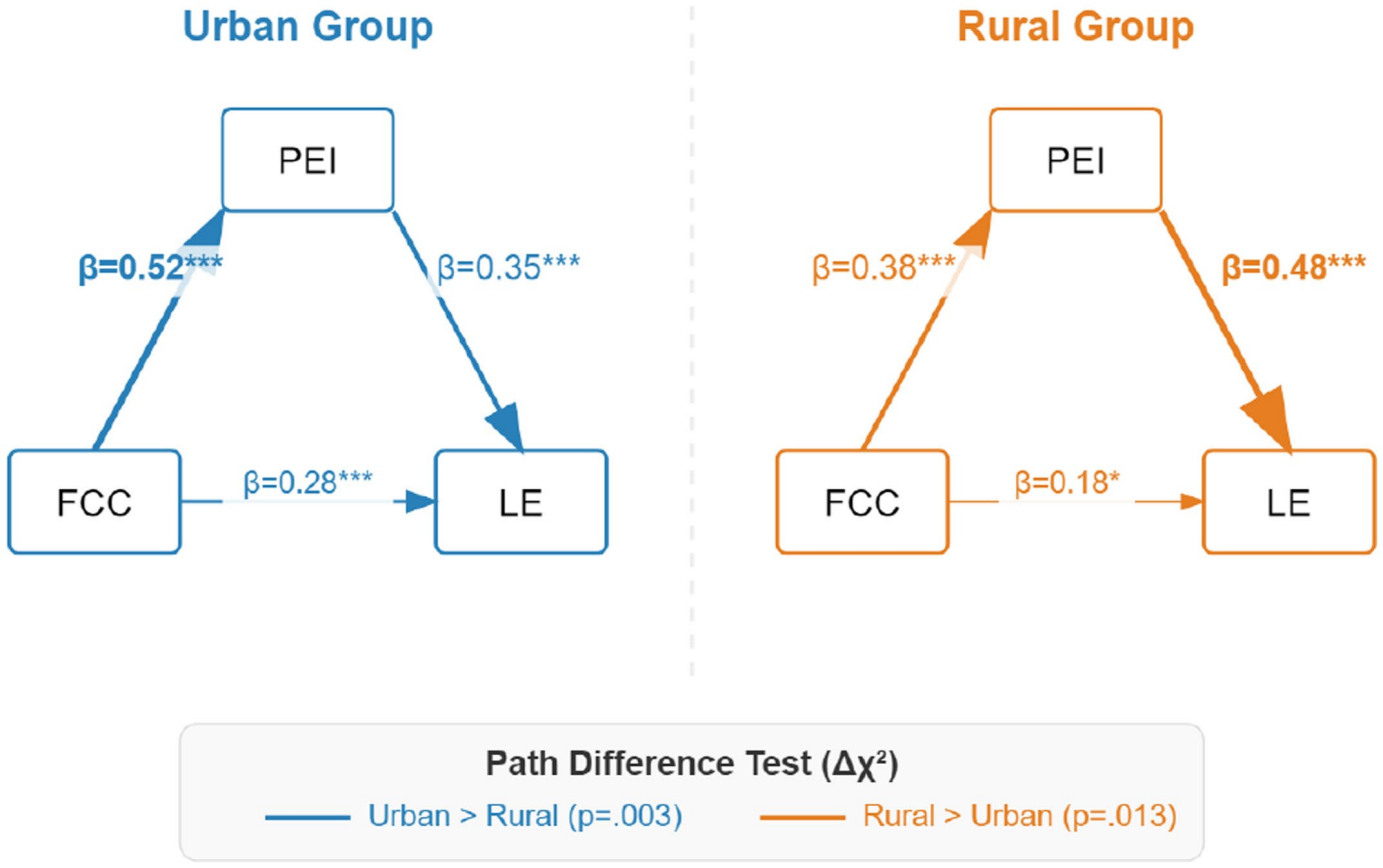
Path coefficients and difference test for urban and rural groups.

Based on established metric invariance, the path coefficients of the two groups were compared. Results are shown in Table 7 and Figure 5.

**Table 7:** Comparison of path coefficients between urban and rural groups.

Path	Urban group (*n* = 190)	Rural group (*n* = 206)	Δ*χ*^2^	*p*
FCC → PEI	0.52***	0.38***	8.67	0.003
PEI → LE	0.35***	0.48***	6.23	0.013
FCC → LE (direct effect)	0.28***	0.18*	3.45	0.063
FCC → PEI → LE (indirect)	0.182	0.182	0.01	0.920

***p < 0.001, *p < 0.05. Δ*χ*^2^ is the chi-square difference test statistic.

Based on the comparison of path coefficients, urban–rural differences significantly moderate the transmission mechanism of cultural capital. Although the total indirect effects of the two groups are comparable (both 0.182), they show “structural divergence” in specific conversion links:

(1) The “Resource Conversion” advantage of the urban group: As shown in the left model of Figure 5, the path from cultural capital to parental educational involvement (FCC → PEI) is significantly stronger in the urban group (*β*_urban = 0.52 vs. *β*_rural = 0.38, *p* = 0.003). This indicates that the “resource endowment” and perfect social support network in the urban environment provide a more efficient channel for converting potential cultural capital into explicit parental involvement behaviors.
(2) The “Subjective Compensation” effect of the rural group: As shown in the right model of Figure 5, the predictive effect of parental educational involvement on student learning engagement (PEI → LE) is significantly stronger in the rural group (*β*_rural = 0.48 vs. *β*_urban = 0.35, *p* = 0.013). This supports the “compensation hypothesis” in resource-scarce contexts: in rural areas with relatively scarce objective educational resources, parents’ subjective agency (i.e., educational involvement) becomes a key “engine” for compensating for external environmental disadvantages and driving student learning engagement.

In summary, the discovery of the urban–rural moderating effect reveals the dynamic nature of the influence mechanism of cultural capital: urban families gain a first-mover advantage through “resource endowment,” while rural families rely more on “behavioral involvement” to protect and improve academic outcomes.

#### 4.4.2 Moderating effect of grade

The correlation analysis in Table 2 suggested that variable relationships might change with grade. To explore this phenomenon deeply, this study used Model 59 in the PROCESS plugin, referencing Hayes (2017), with grade as the moderating variable, to test its moderating effect on the three paths of the mediation model. All predictor variables were centered. The analysis results are shown in Table 8.

**Table 8:** Test of moderating effect of grade on mediation (*N* = 610).

Predictor	Outcome: parental edu. involvement (PEI)	Outcome: student learning engagement (LE)
	*β* (SE)	*β* (SE)
Control variables		
Student gender	0.04 (0.04)	0.09* (0.04)
Only child status	0.02 (0.04)	0.03 (0.03)
SES	0.18*** (0.04)	0.12** (0.04)
Main effects		
Family cultural capital (FCC)	0.49*** (0.04)	0.20*** (0.04)
Parental edu. involvement (PEI)	—	0.42*** (0.04)
Student grade (grade)	−0.16*** (0.03)	−0.11** (0.03)
Interaction effects		
FCC × grade	−0.09 (0.04)*	0.08 (0.03)*
PEI × grade	—	−0.04 (0.04)
*R* ^2^	0.35	0.47
*F*	58.21***	65.12***

Standardized coefficients are reported. ***p < 0.001, **p < 0.01, *p < 0.05. The PEI × Grade path is not significant (*p* = 0.312).

Results show:

First half path moderation significant: The interaction effect of FCC × Grade on parental educational involvement is significant (*β* = −0.09, *p* < 0.05). This indicates that as the grade increases, the strength of family cultural capital predicting parental educational involvement weakens.

Second half path moderation not significant: The interaction effect of PEI × Grade on student learning engagement is not significant (*β* = −0.04, *p* > 0.05). This indicates that regardless of the grade, the promoting effect of parental educational involvement on student learning engagement is stable and does not significantly decrease with the increase of grade.

Direct path moderation significant: The direct interaction effect of FCC × Grade on student learning engagement is significant and positive (*β* = 0.08, *p* < 0.05). This indicates that as the grade increases, the direct influence of family cultural capital on student learning engagement strengthens instead.

To further analyze the significant interaction effects, a simple slope test was conducted as shown in Table 9.

**Table 9:** Simple slope analysis for different grades.

Grade	FCC → PEI (first half)	FCC → LE (direct path)	Indirect effect (FCC → PEI → LE)
Low grade (grade 1)	0.58***	0.12*	0.244 (0.185, 0.312)
Middle grade (grade 2)	0.49***	0.20***	0.206 (0.158, 0.255)
High grade (grade 3)	0.40***	0.28***	0.168 (0.115, 0.225)

***p < 0.001, *p < 0.05.

**Figure 6:**
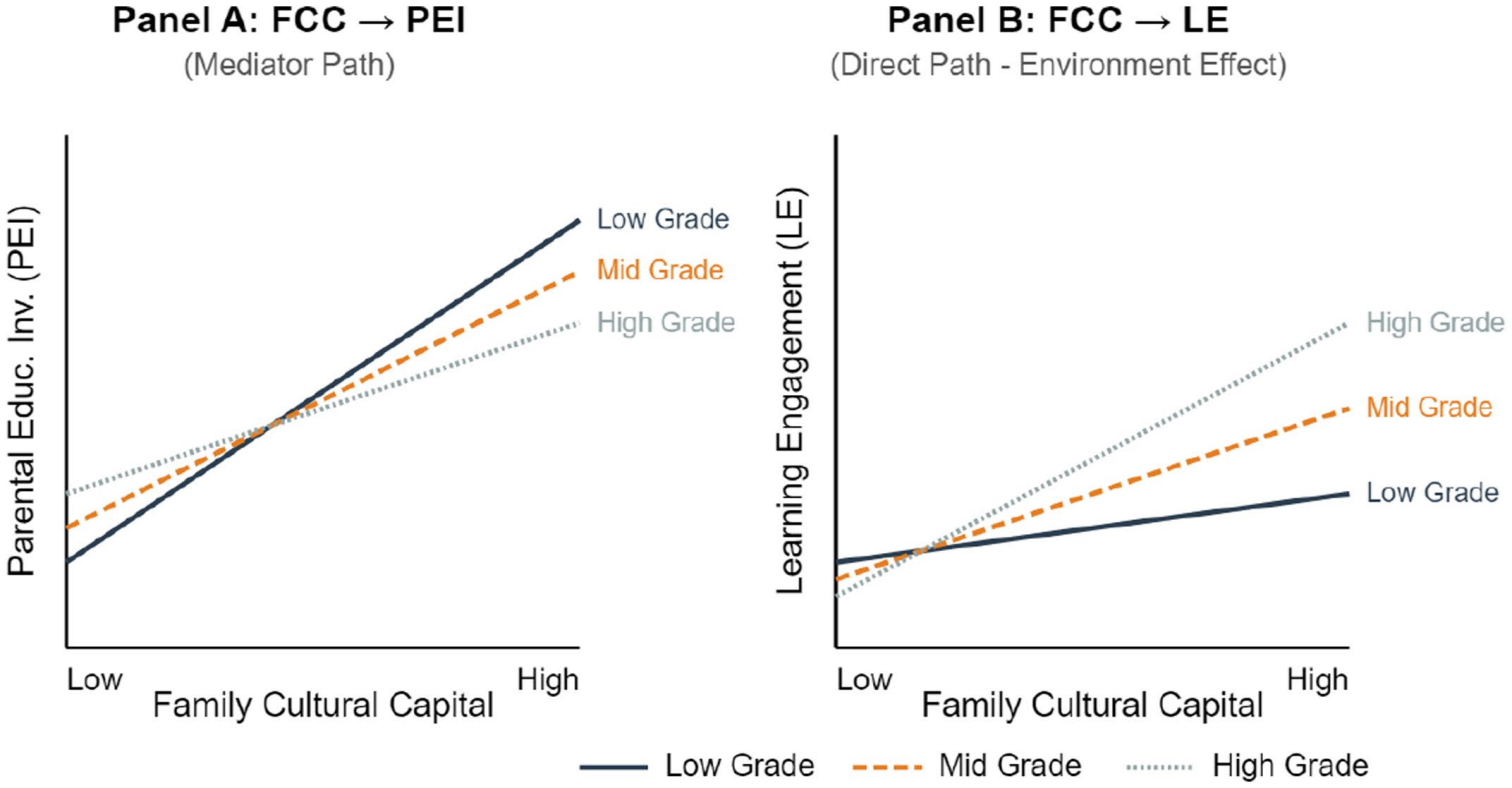
Moderating effect of grade on the action paths of family cultural capital.

To visually analyze the completely opposite moderating trends presented by grade on the “parental involvement mediation path” and the “cultural capital direct path,” a simple slope effect diagram is shown in Figure 6.

Simple slope analysis reveals two distinct trends:

The efficiency of converting cultural capital into parental involvement decreases with grade (0.58 → 0.40). In the lower grades, parents with rich family cultural capital are more inclined to convert it into specific educational involvement behaviors (such as homework tutoring, accompanying reading); while in higher grades, limited by increased curriculum difficulty and enhanced child autonomy, this conversion effect weakens.

The direct impact of cultural capital on learning engagement increases with grade (0.12 → 0.28). In higher grades, although parents’ direct involvement behaviors decrease, family cultural capital (such as book collections, digital resources, cultural atmosphere) directly supports students’ learning engagement in a subtle way. This may imply that higher -grade students are more capable of utilizing family resources for autonomous learning.

Indirect effect weakens with grade. Bootstrap testing shows that the mediating effect of parental educational involvement is strongest in lower grades (Effect = 0.244) and weakest in higher grades (Effect = 0.168). This indicates that in the lower primary school stage, the key to bringing family cultural capital advantages into play lies in “parents getting moving”; while in the higher grade stage, the edification of the family environment itself and resource provision (“environmental presence”) become increasingly important.

### 4.5 Supplementary analysis: the unique role of digital cultural capital

To further verify the unique value of digital cultural capital, a hierarchical regression analysis was conducted. With the cognitive dimension of learning engagement as the dependent variable, control variables and physical cultural capital (book volume, learning space) were entered in the first step, digital cultural capital (digital resource subscription, digital device guidance) in the second step, and community cultural participation in the third step. Results are shown in Table 10.

**Table 10:** Incremental prediction of digital cultural capital on cognitive engagement (*N* = 610).

Predictor	Model 1	Model 2	Model 3
	*β*	*β*	*β*
Control variables			
Student gender	0.09*	0.08*	0.08*
Student grade	−0.18***	−0.16***	−0.15***
SES	0.25***	0.18***	0.16***
Physical cultural capital			
Book volume	0.21***	0.14**	0.12**
Learning space	0.15**	0.12**	0.11*
Digital cultural capital			
Digital resource subscription	—	0.18***	0.16***
Digital device guidance	—	0.22***	0.20***
Community cultural participation			
Museum/library	—	—	0.09*
Community activities	—	—	0.08*
*R* ^2^	0.23	0.31	0.33
Δ*R*^2^	—	0.08***	0.02**
*F*	34.56***	38.92***	35.67***

***p < 0.001, **p < 0.01, *p < 0.05.

The results show that after controlling for physical cultural capital, digital cultural capital still has a significant incremental predictive effect on cognitive engagement (Δ*R*^2^ = 0.08, *p* < 0.001). Among them, the standardized coefficient of digital device guidance (*β* = 0.22) is even higher than that of book volume (*β* = 0.14). This indicates that in the digital age, parents’ guidance on children’s use of digital tools for learning plays an important role in cultivating students’ higher-order thinking skills.

## 5 Discussion

### 5.1 Major findings

This study constructed and verified the mediation model of “Family Cultural Capital → Parental Educational Involvement → Student Learning Engagement.” The core findings are as follows.

Family cultural capital has a significant positive association with student learning engagement. This conclusion supports the applicability of cultural capital theory in the Chinese educational context, consistent with the findings of Xie and He (2023) and Yang et al. (2022). By expanding the measurement of cultural capital to digital resources and community participation, this study more comprehensively reflects the current resource status of contemporary families.

Parental educational involvement plays a partial mediating role between family cultural capital and learning engagement. The 45.1% mediation effect share reveals a potential dynamic pathway for resource conversion: objective cultural resources do not automatically translate into academic advantages; they appear to function through parents’ active guidance. This view resonates strongly with Lareau’s (2018) theory of “concerted cultivation,” which posits that middle-class parents actively foster their children’s talents through organized activities and close monitoring. Our finding provides empirical support for this theoretical claim, demonstrating that parental involvement is a key behavior through which cultural capital is activated. This is also consistent with prior research that identifies parental actions as a crucial link in the family resource conversion chain (Zhang et al., 2020), though few studies have quantified this mediating role in the context of both traditional and digital cultural capital.

Latent profile analysis identified three patterns revealing the heterogeneity of resource allocation. The “Digital Emerging” families, accounting for 43.8%, although inferior to the “All-Round” type in physical resources, performed closely in the cognitive engagement dimension. This suggests that digital cultural capital may offer a path for middle-income families to “overtake on the bend,” helping to narrow educational inequality, which is highly consistent with the significant predictive effect of digital capital on academic achievement found by Jeong et al. (2024) based on PISA 2018 data. This finding contrasts with some earlier studies that expressed concern that digital divides would merely replicate existing inequalities (Roscigno and Ainsworth-Darnell, 1999). Our results suggest a more optimistic possibility: that accessible digital resources, when leveraged effectively, could serve as a compensatory mechanism, a point we will return to.

Urban–rural differences significantly moderate the association patterns. In urban environments, cultural capital is more easily converted into specific parental involvement behaviors; whereas in rural areas, the influence of parents’ involvement behaviors on learning engagement is stronger. This finding supports the “compensation hypothesis,” that is, in resource-poor environments, parents’ subjective efforts can compensate for the lack of objective conditions to a certain extent. Specifically, our results align with Ni et al. (2021) who found a stronger marginal effect of family education behaviors in rural Chinese contexts. The weaker FCC → PEI link in rural areas may reflect a lack of local infrastructure (e.g., museums, libraries) that facilitates the conversion of capital into involvement, whereas the stronger PEI → LE link underscores the heightened importance of parental action when school and community resources are scarce.

Grade differences moderate the path efficiency of the mediation model. The study observed a dynamic evolution process: the efficiency of converting cultural capital into parental involvement decreases as the grade increases, but its direct effect increases with the grade. This finding adds a crucial developmental psychological dimension to the sociological framework of cultural capital. The shift reflects the adaptive nature of parenting in response to a child’s growing autonomy and cognitive maturity. Specifically, the weakening of the mediation path does not imply a withdrawal of parental support, but likely a qualitative shift in involvement forms. As students transition to junior high school, their need for autonomy increases, and the effectiveness of “behavioral involvement,” such as direct monitoring and accompanying reading (which are heavily weighted in the PEI scale), may diminish. This aligns with Self-Determination Theory, which posits that supporting an individual’s sense of volition and choice is critical for their motivation and well-being (Ryan and Deci, 2000). Parents may shift towards “cognitive involvement” (e.g., discussing complex ideas) or “autonomy support” (e.g., providing resources and trusting the child to use them), forms that are more subtle and less captured by traditional involvement measures. This shift explains why the direct environmental influence of cultural capital—the “ambient” effect of a resource-rich home—becomes more prominent for older students, who are better equipped to independently utilize family resources for their learning.

### 5.2 Theoretical contributions

This study expands cultural capital theory in three dimensions.

Expanded theoretical measurement dimensions by incorporating digital capital and community participation. Differing from traditional research that mostly focuses on book volume, this work reflects the diverse forms of family resources in the digital age. Supplementary analysis shows that digital cultural capital has a significant incremental predictive effect on cognitive engagement, and the effect of digital device guidance exceeds traditional book volume, providing quantitative evidence for the development of cultural capital theory in the digital age.

Revealed the potential micro-mediation pathway of cultural capital affecting student development. Traditional research mostly explores the direct association between cultural capital and outcomes. This study introduces parental educational involvement as a mediator, elucidating the plausible dynamic process of converting resources into advantages. The mediation contribution rate highlights the core status of active parental involvement in the process of cultural capital exerting its efficacy, deepening the understanding of the relational patterns.

Identified the spatiotemporal boundary conditions of cultural capital action. Using latent profile analysis and multi-dimensional moderation effect testing, the study confirmed that the efficacy of cultural capital varies by family type, region, and student developmental stage. The identification of “Digital Emerging” families confirms structural changes in capital forms; while the spatial dimension of urban–rural differences and the temporal dimension of grade differences jointly reveal the path regulation law, i.e., lower grades rely on “behavioral mediation” and higher grades are associated with “environmental edification.” The introduction of this dual perspective verifies the dynamic and context-dependent nature of capital action, enhancing the explanatory power of the theory.

### 5.3 Practical implications

The complex mechanism of family cultural capital conversion into learning engagement revealed in this study provides multi-dimensional empirical evidence for family education guidance, home-school governance, and precise reform of public education policies.

#### 5.3.1 Suggestions for parents

Value the accumulation of diverse cultural resources, especially the use of digital resources. Research found that after controlling for physical resources, digital cultural capital still contributes uniquely to cognitive engagement, with the predictive effect of parents’ correct guidance on digital devices exceeding that of traditional book volume. Parents should not be satisfied with just “buying books” but should focus more on “new cultural capital” in the digital age, actively screening high-quality online resources, and guiding children to use digital devices as learning tools rather than entertainment tools.

Implement “stage-based” dynamic parenting strategies. Grade level significantly moderates the action path of cultural capital, so parents should adjust their methods according to their children’s developmental stage. In the lower primary school stage, a “hands-on” strategy should be adopted, activating cultural capital through high-frequency interactive homework tutoring and reading accompaniment. In the upper primary and junior high school stages, a shift to a “resource support” strategy should be made, moving from a direct supervisor to a “resource provider” and “environment creator,” supporting students’ autonomous learning by providing books and digital resources.

Open up the “resource-behavior” conversion chain to activate dormant cultural capital. Parental educational involvement plays a 45.1% mediating role, meaning that displayed books or purchased courses cannot exert maximum effectiveness without interaction. Parents need to convert static resources into dynamic interactions, such as discussing plots together after buying books, exchanging insights after visiting museums, making cultural capital truly convert into learning motivation through parent–child interaction.

Choose differentiated investment paths according to family type. Middle-income families with limited economic capital can refer to the “Digital Emerging” model. Utilizing the advantages of low cost and standardized quality of digital resources, focus on investing in digital cultural capital construction. Through high-quality content and effective usage guidance, achieve breakthroughs in cultivating children’s cognitive engagement.

#### 5.3.2 Suggestions for schools

Strengthen differentiated support for different types of families. Schools should open libraries, provide digital devices, and organize cultural activities for “resource-poor” families to supplement family resource deficiencies. For “Digital Emerging” families, digital literacy training should be provided to assist parents in guiding children more effectively to use digital tools for learning.

Establish a long-term mechanism for home-school cooperation. Parental educational involvement is a key bridge for converting family resources into learning engagement. Through parent meetings, home-school communication platforms, etc., schools should not only feedback on grades but also synchronously improve parents’ ability and willingness to participate in education.

Focus on the multi-dimensional development of student learning engagement. Students from different family backgrounds perform differently in various dimensions of learning engagement. Schools should design teaching activities specifically to balance cultivating learning interest (emotional engagement) and the use of deep strategies (cognitive engagement) while paying attention to students’ daily performance (behavioral engagement).

#### 5.3.3 Suggestions for educational policy

Increase investment in cultural facilities in rural areas. In rural areas, the efficiency of converting cultural capital into parental involvement is low, which is related to the scarcity of public cultural resources. The government should continue to invest in building rural libraries, science and technology museums, and other facilities to provide more community cultural resources for rural families.

Strengthen training and support for rural parents’ educational involvement. The study shows that in resource-poor environments, parents’ subjective efforts have a stronger impact on students. Relevant departments should improve rural parents’ awareness and skills of participation through family education guidance projects, exerting the engine role of the family in adversity.

Promote the popularization and quality improvement of digital education resources. Digital cultural capital is a new path to narrowing educational inequality. The government should increase the development of inclusive high-quality digital resources while strengthening digital literacy training for parents and students to ensure that high-quality education resources can be effectively utilized by families.

### 5.4 Research limitations and future directions

Limited by the research design and objective conditions, this study still has several areas needing improvement. At the level of causal inference, the nature of the cross-sectional survey limits the depiction of the longitudinal evolution trajectory between variables. Although existing theories support the logic of “resources drive behavior,” students’ high level of academic engagement may also generate an arousal effect, inversely motivating parents to increase cultural investment. Subsequent research introducing cross-lagged models or longitudinal designs will help clarify the possible bidirectional interaction characteristics between cultural capital transmission and student development.

The homogeneity of data sources is another limitation. As all key variables were derived from parents’ self-reports, the research conclusions may be influenced by social desirability bias or common method bias. However, the use of parent-reports holds particular ecological validity in this study’s context. Given that a significant portion of the sample consists of lower primary school students (28.5%) whose cognitive capacity for reliable self-evaluation is still developing, parents serve as crucial observers of both family processes and their children’s manifest learning behaviors. Prior research has also indicated a moderate to strong correlation between parent-reported and teacher-reported student engagement, suggesting parents can provide valid assessments of their children’s learning states in the home environment. Nonetheless, to enhance the robustness of the conclusions, future empirical research should aim to construct a multi-subject evaluation system. This would involve integrating students’ self-assessments (especially for older participants), teachers’ objective observations of classroom behavior, and academic achievement records from school systems, thereby using data triangulation to validate the findings.

From the perspective of ecological validity of the sample, existing surveys are limited to economically developed eastern provinces, making it necessary to be cautious when transferring conclusions to central and western regions or families in different social transition backgrounds. The complexity of China’s urban–rural dual structure implies that the conversion efficiency of cultural capital may be deeply moderated by environmental variables such as regional educational infrastructure and social capital density. Subsequent research needs to expand the sampling radius to examine whether digital cultural capital has a “compensation effect” for disadvantaged classes in a broader geographical dimension.

Regarding the excavation of capital connotation, this study currently focuses on the examination of the “quantity” of cultural resources, while relatively ignoring the “quality” in the process of capital use. For example, the screening logic of digital resources, the quality of emotional feedback during parental tutoring, and the depth of interaction in community activities are all core to embodied cultural capital. Future qualitative research or mixed-method research should delve into micro-family scenarios to explore how family resources are converted into students’ endogenous motivation through high-quality parent–child interactions.

Finally, there is room for expansion in the integration of theoretical models. The operation of capital does not exist in isolation; the mutual conversion and game between cultural capital and economic and social capital constitute a complex picture of family education. Future model construction can attempt to introduce interaction terms of multiple capitals and further track their long-term effects on non-cognitive outcomes such as creativity and social–emotional competence, thereby mapping a more inclusive map of family educational resource allocation.

## 6 Conclusion

This study constructed and verified a moderated mediation model of the influence of family cultural capital on student learning engagement, elucidating the micro-mechanism of family resource operation in the digital age.

Empirical results show that the realization of cultural capital efficacy does not rely on simple stacking of resources but highly depends on the pivotal hub of parental educational involvement. Objective resources must be effectively converted into students’ deep learning motivation through parents’ active guidance and interaction. Digital transformation has endowed cultural capital with new connotations, and digital cultural capital shows a scarcity value surpassing traditional physical resources, opening up new channels for improving academic performance for families with relatively scarce resources.

The operational logic of family educational resources presents significant dynamism and heterogeneity in spatiotemporal dimensions. Urban–rural environmental differences reveal the “compensatory participation” characteristics of rural families in adversity. With the increase in school stage, the family influence path undergoes a systematic evolution from explicit behavioral supervision to implicit environmental edification. This evolutionary law reflects that the mode of resource transmission must be adapted to the growth of individual autonomy.

This series of findings deepens the understanding of cultural capital theory in the Chinese context and confirms the potential of digital capital as an emerging force in narrowing the educational gap. Relevant conclusions provide scientific standards for family education guidance, suggesting that parents should pay attention to effective guidance of digital resources and implement dynamic strategies adjusted with school stages. Schools and policymakers should move beyond simple hardware supplementation thinking to precise empowerment at the mechanism level, building a more inclusive educational support system by improving the participation ability and digital literacy of disadvantaged parents, thereby optimizing the realization path of educational equity from the source.

## Data Availability

The original contributions presented in the study are included in the article/Supplementary material, further inquiries can be directed to the corresponding author.
