# Supplementary material for: The influence of family cultural capital on student learning engagement: a study on the mediating role of parental educational involvement
Source: Front Psychol. 2026 Apr 10;17:1802893. doi: 10.3389/fpsyg.2026.1802893 (PMC13105935; doi:10.3389/fpsyg.2026.1802893)
Supplement: Supplementary file 1 [file data_sheet_1.zip › Appendix.docx]

**Appendices**

**Appendix A: Family Cultural Capital Scale Items**

Dimension 1: Physical Cultural Resources

Approximately how many books are in your home? (1=Less than 10, 2=10-50, 3=51-100, 4=101-500, 5=More than 500)

Does your child have an independent learning space? (0=No, 1=Yes)

Dimension 2: Digital Cultural Resources
3. How often do you subscribe to digital learning resources (e.g., online courses, learning apps) for your child? (1=Never, 2=Rarely, 3=Sometimes, 4=Often, 5=Always)
4. How often do you guide your child to use smart devices (e.g., computers, tablets) to look up learning information? (1=Never, 2=Rarely, 3=Sometimes, 4=Often, 5=Always)

Dimension 3: Community Cultural Participation
5. How often do you take your child to visit museums, libraries, and other cultural venues? (1=Never, 2=1-2 times a year, 3=Once a quarter, 4=Once a month, 5=Multiple times a month)
6. How often do you take your child to participate in community cultural activities (e.g., lectures, exhibitions)? (1=Never, 2=Rarely, 3=Sometimes, 4=Often, 5=Always)
7. How often do you utilize surrounding cultural facilities (e.g., science museums, cultural centers) to support your child’s learning? (1=Never, 2=Rarely, 3=Sometimes, 4=Often, 5=Always)

**Appendix B: Parental Educational Involvement Scale Items**

Dimension 1: Home Learning Support

How often do you discuss school situations with your child? (1=Never, 2=Rarely, 3=Sometimes, 4=Often, 5=Always)

How often do you create a good learning environment (e.g., quiet, tidy) for your child? (1=Never, 2=Rarely, 3=Sometimes, 4=Often, 5=Always)

How often do you accompany your child in reading? (1=Never, 2=Rarely, 3=Sometimes, 4=Often, 5=Always)

Dimension 2: Home-School-Community Connection
4. How often do you communicate with teachers about your child’s learning situation? (1=Never, 2=Rarely, 3=Sometimes, 4=Often, 5=Always)
5. How often do you attend school parent-teacher meetings? (1=Never, 2=Rarely, 3=Sometimes, 4=Often, 5=Always)
6. How often do you take your child to participate in social practice activities? (1=Never, 2=Rarely, 3=Sometimes, 4=Often, 5=Always)

Note: Item 5 is an attention check question (“To ensure you are answering carefully, please select ‘Always’ for this item”), and is not included in the total score.

**Appendix C: Student Learning Engagement Scale Items**

Dimension 1: Behavioral Engagement

Your child can complete learning tasks with self-discipline. (1=Completely disagree, 2=Disagree somewhat, 3=Neutral, 4=Agree somewhat, 5=Completely agree)

Your child persists in studying when encountering learning difficulties. (1=Completely disagree, 2=Disagree somewhat, 3=Neutral, 4=Agree somewhat, 5=Completely agree)

Dimension 2: Emotional Engagement
3. Your child is full of curiosity about new knowledge. (1=Completely disagree, 2=Disagree somewhat, 3=Neutral, 4=Agree somewhat, 5=Completely agree)
4. Your child feels enjoyment while studying. (1=Completely disagree, 2=Disagree somewhat, 3=Neutral, 4=Agree somewhat, 5=Completely agree)

Dimension 3: Cognitive Engagement
5. Your child connects new knowledge with existing knowledge in their thinking. (1=Completely disagree, 2=Disagree somewhat, 3=Neutral, 4=Agree somewhat, 5=Completely agree)
6. Your child reflects on their own learning. (1=Completely disagree, 2=Disagree somewhat, 3=Neutral, 4=Agree somewhat, 5=Completely agree)
7. Your child can apply learned knowledge to real life. (1=Completely disagree, 2=Disagree somewhat, 3=Neutral, 4=Agree somewhat, 5=Completely agree)
